# Supplementary material for: Molecular Subtypes Based on Genomic and Transcriptomic Features Correlate with the Responsiveness to Immune Checkpoint Inhibitors in Metastatic Clear Cell Renal Cell Carcinoma
Source: Cancers (Basel). 2022 May 10;14(10):2354. doi: 10.3390/cancers14102354 (PMC9139776; doi:10.3390/cancers14102354)
Supplement: Supplementary file 1 [file cancers-14-02354-s001.zip › cancers-1703346-supplementary.pdf]

cancers

MDPI

Supplementary Materials

Molecular Subtypes Based on Genomic and Transcriptomic Features Correlate with the Responsiveness to Immune Checkpoint Inhibitors in Metastatic Clear Cell Renal Cell Carcinoma

ByulA Jee [1], Eunjeong Seo [1], Kyunghee Park [2], Yi Rang Kim [3], Sun-ju Byeon [4], Sang Min Lee [1], Jae Hoon Chung [1], Wan Song [1], Hyun Hwan Sung [1], Hwang Gyun Jeon [1], Byong Chang Jeong [1], Seong Il Seo [1], Seong Soo Jeon [1], Hyun Moo Lee [1], Se Hoon Park [5], Woong-Yang Park [2] and Minyong Kang [1,2,6,*]

[1] Department of Urology, Samsung Medical Center, Sungkyunkwan University School of Medicine, Seoul 06531, Korea; astherjee@skku.edu (B.J.); ejseo09@skku.edu (E.S.); s2623.lee@samsung.com (S.M.L.); jaehoontasker.chung@samsung.com (J.H.C.); wan.song@samsung.com (W.S.); hyunhwan.sung@samsung.com (H.H.S.); hwanggyun.jeon@samsung.com (H.G.J.); bc2.jung@samsung.com (B.C.J.); seongil.seo@samsung.com (S.I.S.); seongsoo.jeon@samsung.com (S.S.J.); hyunmoo.lee@samsung.com (H.M.L.)

[2] Samsung Genome Institute, Samsung Medical Center; Seoul 06531, Korea; kyunghee.park@samsung.com (K.P.); woongyang.park@samsung.com (W.-Y.P.)

[3] Oncocross Ltd., Seoul 04168, Korea; 99yirang@oncocross.com

[4] Department of Pathology, Hallym University Dongtan Sacred Heart Hospital, Hwaseong 18450, Korea; byeon.sunju@welovedoctor.com

[5] Division of Hematology-Oncology, Department of Internal Medicine, Samsung Medical Center, Sungkyunkwan University School of Medicine; Seoul 06531, Korea; sh1767.park@samsung.com

[6] Department of Health Sciences and Technology, The Samsung Advanced Institute for Health Sciences & Technology (SAIHST), Sungkyunkwan University, Seoul 06355, Korea

* Correspondence: m79.kang@skku.edu

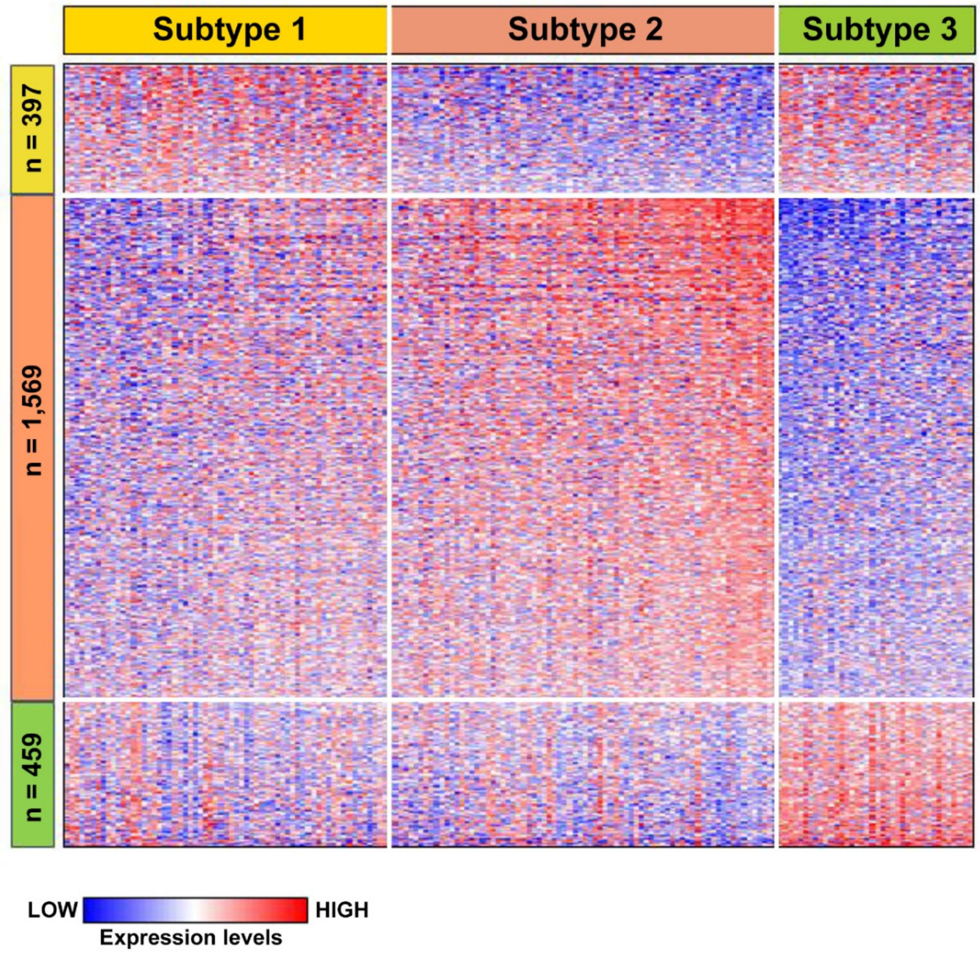

Figure S1. A heatmap showed up-regulated genes in each subtype.

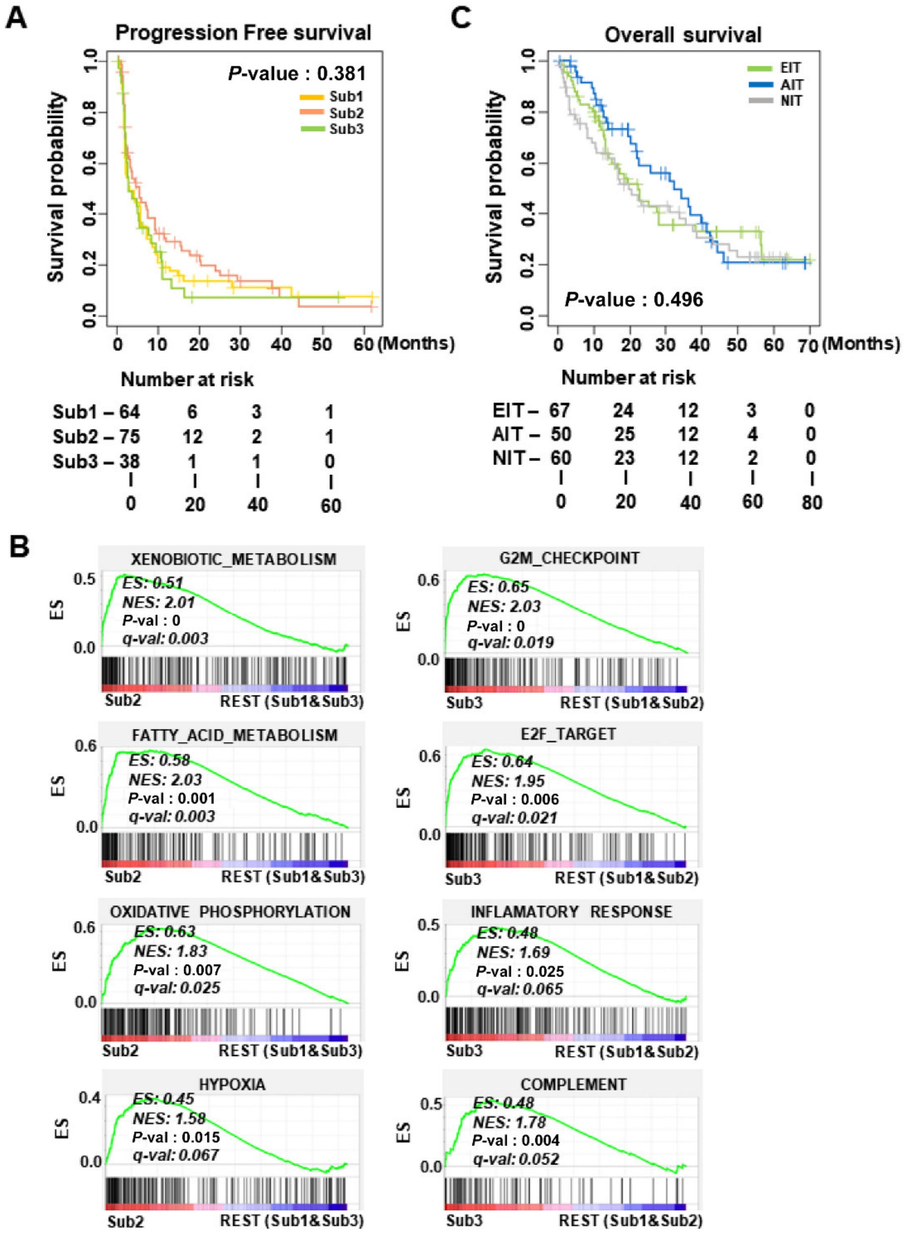

Figure S2. Kaplan-Meier plots for PFS and OS and ssGSEA analysis using HALLMARK gene sets. (**A**) Kaplan-Meier plot analysis of progression free survival (PFS) for each subtype. Sub1, Subtype 1 (yellow); Sub2, Subtype 2 (pink); Sub3, Subtype 3 (green). (**B**) GSEA analysis using HALLMARK gene sets (ES, Enrichment score; NES, Normalized enrichment score) (**C**) Kaplan-Meier plot analysis of overall survival (OS) for each immune type. AIT, Active-immune type (navy); EIT, Exhausted-immune type (green); NIT, Non-immune type (gray).

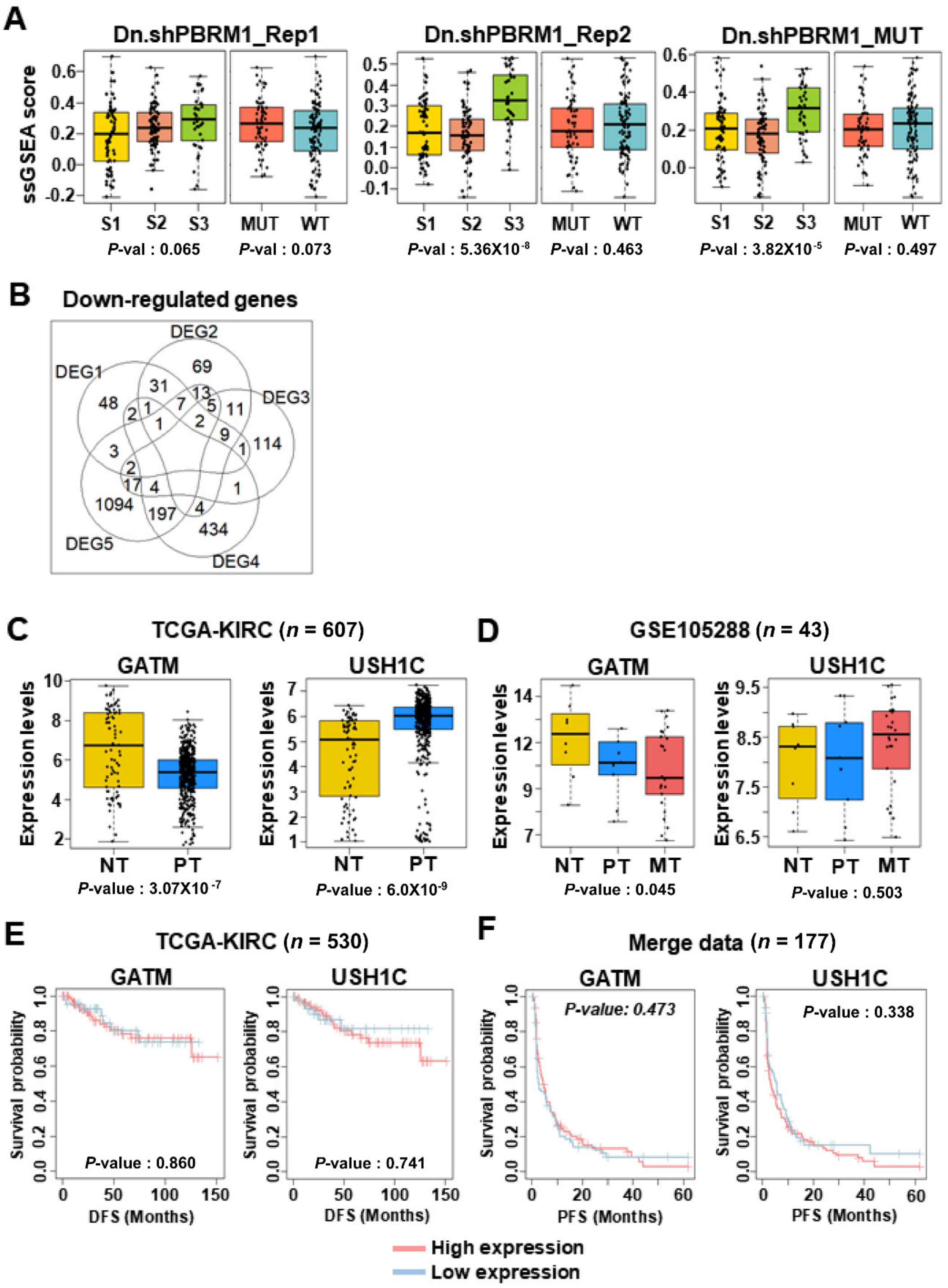

Figure S3. Kaplan-Meier plots and boxplots based on *GATM* and *USHIC* expressions. (A) Boxplots summarizing the heatmap in Figure 4A in each subtype (*left*) and in group with *PBRM1* mutation vs. group without *PBRM1* mutation (*right*), respectively. (S1, Sub1; S2, Sub2; S3, Sub3; MUT, *PBRM1* mutation; WT, *PBRM1* wild type) (**B**) Venn diagram showed the overlapped down-regulated 5 DEGs. (**C**) Boxplots showed the expression levels of *GATM* and *USH1C* in TCGA-KIRC. NT, non-tumor (*n* = 77); PT, primary tumor (*n* = 530) (**D**) Boxplots showed the expression levels of *GATM* and *USH1C* in GSE105288. NT, non-tumor (*n* = 8); PT, primary tumor (*n* = 9); MT, metastasis tumor (n = 26). (**E**) Kaplan-Meier plots showed disease free survival (DFS) for the patients group stratified

by above or below the average *GATM* (*left*) or *USH1C* (*right*) in TCGA-KIRC (*n* = 530). High expression (pink); low expression (blue). (**F**) Kaplan-Meier plots showed progression free survival (PFS) for the patients group stratified by above or below the average *GATM* (left) or *USH1C* (right) in the merge data (*n* = 177). High expression (pink); low expression (sky blue).

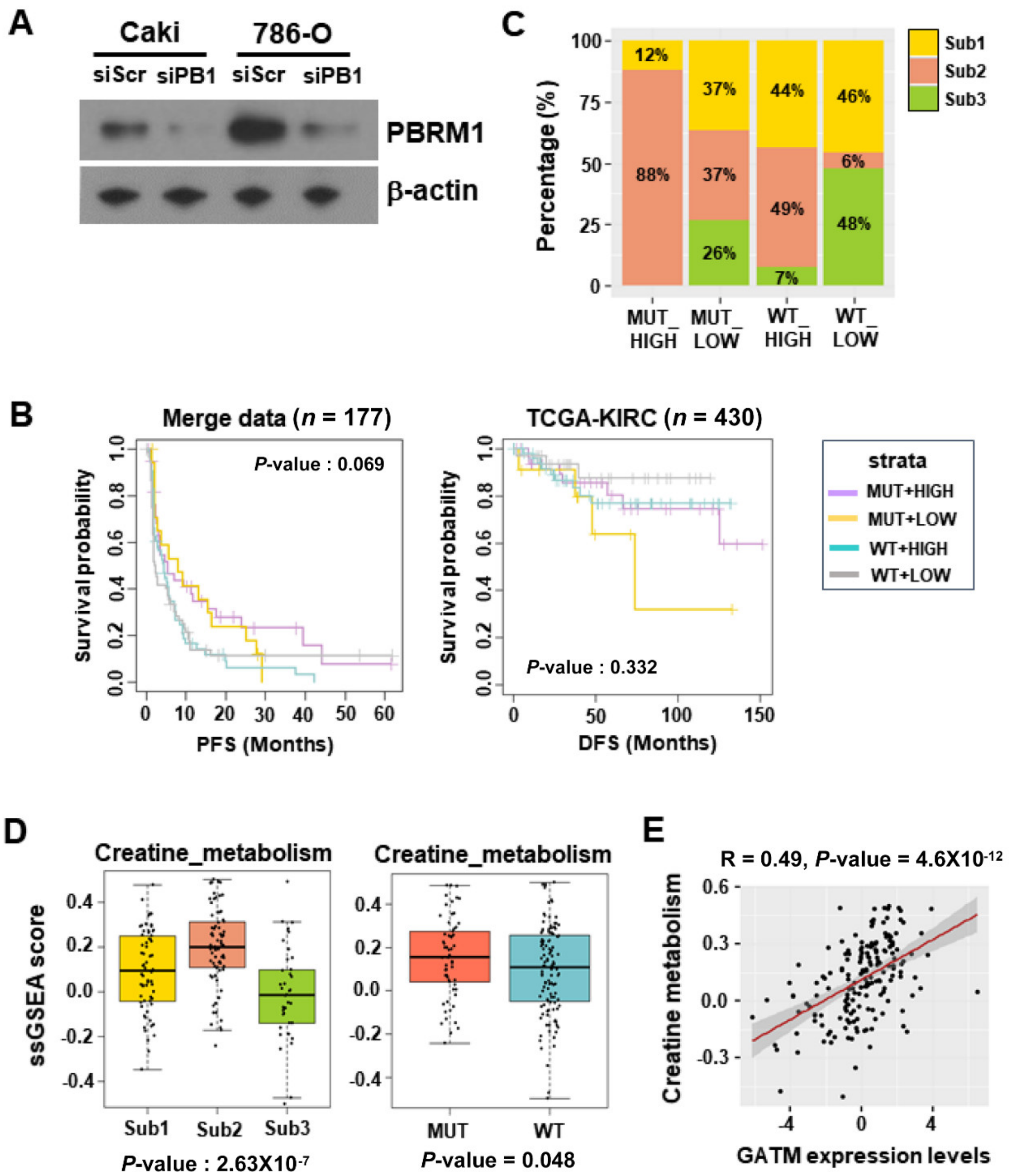

Figure S4. Kaplan-Meier plots based on *PBRM1* mutation and *GATM* expression and correlation plot between creatin metabolism and *GATM* expression. (A) 786-O or Caki-1 cells were transfected with siScr or siPBRM1 and PBRM1 protein level was measured by western blot. (B) Kaplan–Meier plot analysis of progression-free survival and disease-free survival based on *PBRM1* mutation and *GATM* expression. PBRM1_MUT+HIGH_GATM (*n* = 42, purple), PBRM1_MUT+LOW_GATM (*n* = 19, yellow), PBRM1_WT+HIGH_GATM (*n* = 55, blue), and PBRM1_WT+LOW_GATM (*n* = 61, gray). Kaplan–Meier plot analysis of disease-free survival based on *PBRM1* mutation and *GATM* expression using TCGA-KIRC. PBRM1_MUT+HIGH_GATM (*n* = 92, purple), PBRM1_MUT+LOW_GATM (*n* = 46, yellow), PBRM1_WT+HIGH_GATM (*n* = 161, blue), and PBRM1_WT+LOW_GATM (*n* = 131, gray). (C) Barplot showed the percentage of subtypes in the four groups (MUT_HIGH, PBRM1_MUT+HIGH_GATM; MUT_LOW, PBRM1_MUT+LOW_GATM; WT_HIGH, PBRM1_WT+HIGH_GATM; WT_LOW, PBRM1_WT+LOW_GATM) (D) Boxplots showed ssGSEA

scores for the signature of creatine metabolism in each subtype (*left*) and in group with PBRM1 mutation vs. group without PBRM1 mutation (*right*), respectively. (S1, Sub1; S2, Sub2; S3, Sub3; MUT, *PBRM1* mutation; WT, *PBRM1* wild type) (E) Correlation plot between creatine metabolism and *GATM* expression levels.

Table S1. Baseline clinical characteristics of patients with metastatic renal cell carcinoma (n = 60).

Characteristic	Value
Age (yr), median (IQR)	56.5 (18–84)
Sex	
Male	43 (72%)
Female	17 (28%)
Heng risk group	
Favorable	4 (7%)
Intermediate	40 (67%)
Poor	16 (26%)
ECOG performance status	
0–1	56 (93%)
2	4 (7%)
No. of metastasis	
Single	18 (30%)
Multiple (≥2)	42 (70%)
Site of metastasis	
Lung	49 (82%)
Lymph node	25 (42%)
Bone	25 (42%)
Liver	8 (13%)
Prior nephrectomy	52 (87%)
Type of systemic therapy	
1st line	8 (13%)
2nd line	52 (87%)

Note: Values are presented as number (%), unless otherwise indicated. Abbreviations: ECOG, Eastern Cooperative Oncology Group; IQR, interquartile range.

Table S2. Baseline outcomes between patients receiving first-line and second-line therapies.

Variables	First line (n = 8)	Second line (n = 52)
Outcomes		
IMDC risk (N, %)		
Favorable	**0 (0.0)**	**4 (7.7)**
Intermediate	**4 (50.0)**	**36 (69.2)**
Poor	**4 (50.0)**	**12 (23.1)**
ORR (N, %)		
CR	**0 (0.0)**	**3 (5.8)**
PR	**5 (62.5)**	**13 (25.0)**
SD	**0 (0.0)**	**7 (13.5)**
PD	**3 (37.5)**	**25 (48.1)**
Unknown	**0 (0.0)**	**4 (7.7)**
Benefit (N, %)		
CB	**5 (52.5)**	**16 (30.8)**
ICB	**0 (0.0)**	**5 (9.6)**
NCB	**3 (37.5)**	**28 (53.8)**
Unknown	**0 (0.0)**	**3 (5.8)**
Progression-free survival Median (range), months	**6.4 (2.2–12.8)**	**2.9 (0.0–43.9)**
Overall survival Median (range), months	**8.4 (3.6–14.8)**	**10.7 (0.0–43.9)**

Abbreviations: IMDC, International Metastatic RCC Database Consortium; ORR, objective response rate; CR, complete response; PR, partial response; SD, stable disease; PD, progressive disease; CB, clinical benefit; ICB, intermediate clinical benefit; NCB, nonclinical benefit.

Table S3. Upregulated genes in each subtype.

Subtype 1	Subtype 2	Subtype 3
ONECUT2	GSTA2	RRP9
MAT1A	SLC5A12	TUBB6
MAGED4	ACSM2A	TRIM25
TNNT2	GSTA1	SARNP
LCN2	ACSM2B	CCDC88A
SLPI	SLC6A13	PRKDC
IL2RA	DDC	ELP6
TM4SF4	LRP2	PSMB7
DMP1	GLYAT	MOSPD2
TP53TG3	SLC5A8	VIMP
KRT16	SLC6A3	ARPC2
GREM1	TMEM174	ZYX
LAD1	SLC17A3	PFN1
ARHGAP40	AGXT2	EMILIN2
ASIC1	SLC5A1	ETV3
ARL14	TMEM27	ARF1
PAEP	NAT8	COTL1
ANKRD1	SLC22A6	ATAD2
ORM1	ACE2	ACTB
COL7A1	SLC5A10	PHTF2
GOLGA6L2	TRHDE	ARPC5L
ERC2	FUT6	TCF3
PI3	CREB3L3	ELK3
NTNG1	KHK	PLOD3
CYP2C18	SLC47A1	ARL6IP1
PLA2G4F	SLC22A2	BCL10
PRKCG	FMO1	FAM219A
DUX4L4	MIOX	FAM114A1
KIR2DL4	HNF4A	KIAA1524
TSPAN8	UGT1A9	FGD6
KIAA1211	PRODH2	PSMA7
KRT14	RNF186	NUDT19
KRT6A	BBOX1	PLEC
CEACAM6	HAO2	GNAS
SP5	SLC28A1	PSMA5
DCAF12L2	DPYS	GNAI2
CPEB1	SLC17A4	CDV3
B3GAT1	CYP4A11	PABPC1
SCN3A	SLC13A1	ARPC5
PRSS22	A1CF	EXT1
POF1B	MGAM	PRKCD
HCAR2	HHLA2	PDXK
SFN	SCGN	CD47
SFTA2	BHMT	SEC13
PODNL1	PKLR	TWF2
PKP3	SLC39A5	BZW1
GPRC5A	KCNJ3	TUBB4B
CHST6	TM4SF5	NIPSNAP3A
KCNG1	ASPG	CFL1
IL31RA	CLRN3	GPN1
FAXC	SLC6A18	CHPF2
FFAR1	DAO	TGFBR1
FLG	SLC22A11	PEX16
COL6A5	SLC10A2	KIAA0930
IGFN1	AMN	GSTO1
AGR2	CUBN	BMP1
SLC8A2	CES3	PLXNA3

LMOD3	SLC16A12	C11orf24
TSPY8	UPB1	RCC2
SMPX	AZGP1	PLA2G15
FAM83B	PAH	ARL8A
LAMC2	GLYATL1	GNG5
SNTG2	SLC16A9	H2AFZ
BCAS1	DOC2A	C7orf73
GSTM5	SERPINF2	ELOVL1
FA2H	SLC22A12	KCTD11
SUSD4	CLDN10	CAPZB
GLDN	ACMSD	GNL3
PTX3	ENPP3	GARS
FAM169A	PRAP1	AIMP2
FCRLA	HPN	SLC52A2
COL22A1	ANKS4B	PSMA4
ADAMTSL5	AGMAT	DYRK4
IGF2BP2	MLXIPL	CCR1
CCL11	TRPM3	MARCKSL1
STK33	CLEC18A	TUBA1A
KLHL10	SLC17A1	FBXO5
RP11-404P21.8	UGT2A3	KDELR2
KLK11	CYP24A1	LSM1
TUBB2B	PKHD1	RRAGC
DRC1	USH1C	MAN2B1
GDNF	C1orf210	SH3PXD2A
STK32A	SERPINA6	PPIB
TFAP2C	TRIM15	ADAM19
C11orf45	GATM	VHL
MMP23B	ACADL	SAMHD1
FAM3B	WDR72	MALSU1
SLCO5A1	SLC22A24	TMED2
SYT3	ACY3	FKBP1A
DCC	ASPA	CENPO
BDKRB1	CABP1	MPZL1
ETV4	SLC3A1	MARVELD1
ADAMDEC1	NPR3	ELF4
DMRT2	SLC7A9	ANKRD52
THSD4	LGALS2	ARID3A
CECR2	CLDN2	TMSB4X
HOXB13	FXYD2	TRAM2
B4GALNT3	GRIA4	PBX3
WNK2	PDZK1	SH3BGRL3
LYPD1	PCK1	TACC3
KRTAP10-1	CYP2J2	GNB4
PIGZ	TRIM10	HELZ2
P2RX5	ALDH1L1	MYOF
OLFM4	ACSM5	C19orf48
CR1	HNF1A	GALNT1
PI15	EHHADH	YKT6
SEMG2	SLC2A2	CTSZ
PRTG	FREM2	MYO5A
TFR2	ALDH8A1	CAMK1D
NIPAL4	G6PC	LAP3
GPR45	FAM150B	TAGLN2
PPM1H	EPHA7	ARPC3
NEFH	MOGAT3	ALYREF
ADAM23	CHST9	TMEM104
SLC6A4	ARSE	COLGALT1
C1orf226	GIPC2	CNN2
TACSTD2	TMEM252	LRP1

FPR2	REG1A	DSCC1
STRA6	AGMO	LSM7
PKNOX2	CRYM	NT5DC2
LAMB3	SLC27A2	IRAK1
CD22	ESPN	NAV1
ZNF726	CDHR5	AXL
IL24	PHYHIPL	TRIAP1
LINC00346	ARSF	S100A16
EPGN	SYT9	MANF
TRIM29	RBP5	FXYD5
RGPD1	APOM	ECT2
FAM129C	PDZD3	GLIPR2
DUOXA2	EMX2	CDCA4
PSG9	SLC25A48	PSMD14
TENM3	TINAG	FPR3
NKX2-1	AQP4	ANXA1
HOMER2	SCGB1D2	STIL
PLAC8	PLA2G12B	SLC25A19
C6orf141	COL23A1	HAUS6
SERPINI2	ERBB3	LCP2
ACTBL2	GDA	TIFA
ISLR2	CLEC18C	DBF4
OR5P3	FAM153C	RGS19
A1BG	ADCY5	APRT
SCN2A	CMBL	ARPC1B
ZNF648	FABP7	TIMP2
FZD10	SULT1C4	GPX1
DSPP	CLEC18B	SHCBP1
APBA2	SLC6A12	TUBA1B
PIANP	MAOB	RHOG
MEI1	GPT	MAFB
GDF5	TMEM82	TGFB1
SLC7A11	BHMT2	UBE2S
GUCY2D	HMGCS2	NRP2
F3	RP11-6L6.2	SH3GL1
HSD11B1	ASXL3	PGAM5
ISL2	UGT1A6	GMIP
MUC12	HIST1H2BA	ADCY7
KCNG3	AMDHD1	CSF1
HMGA1	GJB1	CHCHD2
GJB6	CCDC178	SQRDL
PTGES3L	ANXA13	SPI1
C7orf69	LYG1	CTSB
TNFRSF18	AC106876.2	SERPINH1
SIX4	RGN	IKBIP
FOXG1	MASP1	BRCA2
PRAMEF19	FOXB2	TRPM2
MUC16	RERG	ACP2
TFCP2L1	ANO3	CMTM3
PTPRQ	PDK4	TAF1A
ANKFN1	SLCO4C1	RALA
CLIC3	DMGDH	ECM1
SLC22A31	RAB17	GLA
CREB3L1	TEX15	ETV5
C3orf52	SLC6A19	HCK
FRMD5	DNAI1	S1PR2
SNCAIP	UGT1A7	TICRR
CTSV	CYB5A	TMSB10
ITIH3	PTGER3	MTAP
CSHL1	ECHDC3	GPR173

LPAR3	KCNJ15	RRM1
PLTP	TENM1	FERMT3
PANX2	PTH2R	LAPTM5
PDGFRA	CAPN13	CD53
DUOX2	MAPT	RAP2B
KRT9	GABRB3	LRRC25
POTED	FTCD	ITGB2
GFPT2	GPD1	TUBA4A
RORB	MGARP	PPP1R14B
SPEG	RP11-407N17.3	ZNF738
HS6ST2	RUNDC3B	LIMD1
TRNP1	AOC1	KIRREL
OTX1	ADSSL1	RAB31
GDF1	VIL1	FANCI
SLAMF7	ALDOB	SLC2A6
EPS8L1	NR1H4	PLEK
HPDL	GRIK3	RACGAP1
LRP8	TRIM55	VIM
HHIPL2	CXCL14	ZNF93
MAGEC1	ANO4	TUBB
IRS4	ACAD11	RUNX1
DYDC2	MIA2	LGALS9
NAV3	AOX1	PLB1
C4orf50	UGT2B11	CLIC2
TCL1A	RP11-10A14.4	ARF4
TNFAIP8L3	DIRAS2	C3orf62
RIMS3	TMEM171	CMKLR1
PMAIP1	UGT2B7	CENPE
CDH10	ABCC6	CCL1
MTUS2	GPX3	FBN1
CLCA2	GYPA	CKAP4
BFSP1	KLHL32	SDC3
RGAG1	TAL2	RNASEH2A
CLEC4C	RIMKLA	BUB1
MOXD1	QRFPR	MX2
SLC52A1	C14orf105	XRCC2
FSCN2	ADIRF	UCK2
FRMD7	NPY	PFDN4
KIF2B	CRB3	FSCN1
CD163L1	MLIP	PLK1
NAP1L2	PANK1	QSOX1
WNT5A	ENPEP	OSCAR
DOC2B	PPARGC1A	CMSS1
C16orf54	PALM3	FOXM1
GDF5OS	GAL3ST1	ADAMTS14
KRT72	KLF15	PLP2
HAS2	MAP7	SLC15A3
NXPH2	OR2T10	PARPBP
FRMPD3	ABLIM3	CKLF
CNGA3	AKR7A3	SDCBP
FAHD2B	C11orf52	NCAPH
ALS2CL	TSPAN12	CD33
UTF1	VEGFA	AIF1
WDR87	DNAH11	FZD2
RSPH4A	DSCAML1	CALU
C10orf90	SGSM1	IQGAP3
PDE6A	PBLD	SLC31A1
GPR156	IMPA2	YIF1A
PLAG1	AGT	SH3PXD2B
ARL4D	SERPINA1	CLPSL2

POTEI	SMLR1	LPXN
AXIN2	BAIAP2L2	LAIR1
KIF26B	SLC22A7	COL6A2
ISLR	TMEM37	KIF18B
HSH2D	OR2T5	DOK2
C17orf51	EVPLL	IL4I1
GPR84	CNDP1	KIF11
ZNF80	KCNJ16	PROCR
MYO10	ANGPTL4	MSR1
SLC29A2	FCAMR	CECR1
APCDD1L	CYS1	RP11-432B6.3
HOPX	SLC23A3	TSPO
CXCR5	C11orf54	DLGAP5
MCOLN2	PAX2	EMP3
LAMP3	SOWAHB	UCP2
OR10G4	MYOT	ARHGAP11A
SEMG1	KCNK9	GAL3ST4
CEACAM7	CES4A	ARL4C
MAFA	AR	SIGLEC9
TRIML2	PRUNE2	LILRA6
SLC5A5	CASC1	NOP10
OR10H1	IQUB	KIF23
OR10G8	KL	BLM
COL4A6	HLF	CCNA2
TSPYL5	ASRGL1	APOL4
IGFBP2	MAOA	PLXNA1
PFKFB2	IL17RB	ASPM
SLC35G3	TMEM72	CD276
SLITRK3	CASQ2	OSTC
KCND1	PLIN2	GPX8
CLIP4	TLR3	TMEM158
LOR	POU5F1	CENPK
TGFB2	ENTPD8	NBL1
ENTHD1	ALPL	IL7
SLC25A35	SALL1	S100A6
ZKSCAN4	SEMA5B	FN1
PRKCB	CAPN12	FBLN1
HCN4	SLITRK4	NCAPG
CHD3	TCEA3	MMP14
PODN	ESRRG	LILRB4
PAQR8	RNASE4	AAED1
MCIDAS	EPHX2	RGS10
F13A1	CDH16	TUBB2A
LCE5A	FABP6	IGFBP6
CUX2	GAMT	COL6A1
PSAPL1	ESM1	HAPLN3
SLC1A6	DNAJC22	RFC4
TYRO3	GGT1	SECTM1
GSG2	PPP1R14D	MMD
RAB3B	CRIP3	MRC2
PYGO1	GALR1	MKI67
SLC24A3	C1QL4	RIPK2
EXD1	UGT1A3	CDC6
COL8A2	ATP13A4	ASF1B
OR10G2	MYO3A	KIFC1
SATB1	TEX11	CKAP2L
TMEM132A	NDUFA4L2	KIF15
SMARCD3	GOLGA8M	CDC25C
RANGRF	ABCC2	IL1RAP
AKAP5	RP1-127H14.3	FBN2

GCNT1	AQP3	COL1A2
SEMA7A	HSD3B2	POLR2J
LRR1	SLC25A52	CILP
HS3ST3B1	HSF4	COLEC12
HAS3	ALDOC	BIRC5
ZNF239	BARX2	LILRB1
OR6C3	NRTN	CD82
FGD1	SLC4A4	KIF14
ARHGEF26	SORBS2	AURKA
LRFN1	GALNT14	KPNA2
MAP3K9	GLIS1	NDC80
FBLIM1	HAVCR1	CDC20
PSKH2	PNCK	IFI27
SV2A	SLC17A2	PLK4
GAS7	OVOL1	ANXA2
WFIKKN2	RAP1GAP	MMP2
WDHD1	MYO7B	TTK
CASZ1	CXorf57	BASP1
CD44	PHKA2	LGALS1
RALGPS1	MPV17L	OASL
LRRC52	YBX2	ALOX5AP
PDX1	TMIGD1	HCST
ALDH1L2	HILPDA	ORC1
GALNT7	IFNA5	KDELR3
LRFN2	OTOGL	GXYLT2
OR4C3	SLC2A4	SIGLEC1
CHIA	ETNK2	TNFRSF8
NXPE3	OR2T35	KIF4A
SNPH	BTNL9	DRP2
MAN1C1	CCL28	NT5E
CCER1	OR2T34	GPR68
XKR6	KCNIP4	CCNE1
ZNF716	HRASLS2	CENPF
DOCK3	NLGN1	CTSS
DACT1	AFM	EXO1
ABCD2	COLCA1	SKA1
DBN1	SHMT1	SEMA3C
MFHAS1	FAM151A	CYBB
DNAH2	FOLR1	TYROBP
CCDC136	EXOC3L4	KIF20A
OTOP2	KLKB1	MAD2L1
TSHZ3	COX4I2	CCNB2
MTHFD2	KCTD14	CD14
MEX3B	COL4A3	COL5A2
SPTB	PGF	S100A11
HIGD1A	ELOVL7	C14orf132
TMC6	TMEM38B	HIST1H3D
COL16A1	RERGL	SOX1
TET1	PDGFD	BCAT1
BAIAP2L1	FUT3	MMP11
RP11-766F14.2	SLC5A9	TBC1D2
SIGLECL1	ZNF704	FAM64A
OR4A16	TRIM6	SIGLEC7
CEP128	LECT2	CDCA8
TEX35	MYL3	CTHRC1
ZIK1	EGLN3	COL3A1
LRFN3	RP11-1026M7.2	CDK1
BTK	GHR	COL6A3
LRFN4	CACNA1E	COL5A1
KLHL18	CDHR3	C1QC

KCNH5	WDR93	HIST1H2AG
WNT7A	MAPK15	SLAMF8
NUMBL	MFAP3L	PDCD1LG2
TMPRSS11E	CA4	FOSL1
TLE4	DEFB1	IFI30
CHAF1B	CDHR2	PLA2G7
SUSD1	CRYL1	KIF2C
KCTD1	MYRFL	CRYBB1
GNA15	ZMYND12	IFI6
OR5M8	REPS2	HIST1H1B
LYN	OR51M1	SYN1
MICAL2	SLC38A11	CDCP1
HEATR5A	TSPAN7	CERCAM
METRNL	SLC2A5	FNDC1
ZNF469	DEPDC7	ZWILCH
CDR2L	KCNE3	CKS2
OR6N2	SORCS1	IGDCC4
AP4B1	CLVS2	HN1
OR5M9	FBXO17	CDCA5
NUP210	NEK5	RRM2
NBEAL2	CNDP2	RSAD2
PRR23B	CLCN5	CSTA
SRPK1	ANG	F13B
CCT8L2	EDN1	CENPM
CCNJ	CD70	GHSR
TKTL2	ANPEP	MXRA5
ARHGEF25	HAAO	ANLN
GUCA2A	EMX1	GPNMB
ARHGEF3	TREX2	FCGR1A
POMGNT2	OR2T3	PDGFRL
KLHL25	ATP1B2	KIAA0101
TRERF1	TMEM220	CAMK2N2
FGFBP1	ADM2	PRR11
OR8D1	STK32B	MYBL2
ABR	IRX3	CMPK2
DST	UGT1A5	CD86
ZNF703	CHDH	FAP
MINA	SYPL2	TOP2A
FLJ45079	C21orf62	TENM2
SETMAR	RGS7BP	HIST2H3C
	CA9	AC092675.3
	TMEM176A	CTSK
	MAST4	NUF2
	VWC2L	FCGR3A
	MUC20	HIST1H2AD
	MME	PLAUR
	ADRA1B	LAG3
	HSD11B2	TREM2
	ZNF541	COL1A1
	CYP3A7	HIST1H2BJ
	NPTX2	TPX2
	SMIM2	LOXL1
	UGT1A4	ATP8A2
	ALDH1A1	PLAU
	ATP2B2	SFRP4
	PLCZ1	PLEKHG4B
	GLB1L	SLC38A5
	METTL7B	CHRFAM7A
	NME5	ADAM12
	ACR	C1QB

	TRPV4	HIST1H3J
	TMEM91	C1QA
	F2RL1	LY96
	DHDH	ISG15
	PNPLA7	MMP9
	WDR17	C16orf95
	HNF4G	ZP1
	PLS1	CDC25A
	HCG27	APOC1
	DMRTA1	MILR1
	COL25A1	WNT2
	FRMD3	PYCR1
	NLRP11	MAGED4B
	METTL7A	MELK
	MAATS1	DCBLD2
	STH	FCER1G
	SLC37A4	TFPI2
	B3GNT3	CCL8
	TM6SF2	TWIST1
	LIX1	FAM72B
	C10orf126	UBE2C
	SLC22A5	NCAM2
	THSD7B	EPYC
	COLCA2	ANKRD22
	SLC1A1	APOA2
	CP	EGFL6
	KIF12	HOXB9
	CCDC146	CXCL10
	SMTNL2	MFAP2
	AQP7	MARCO
	PLIN5	CCL13
	CYP3A5	LHX2
	MYOC	CRABP2
	PCOLCE2	LRRC15
	MCF2L	HIST1H2AI
	ABCB1	BNC1
	CRYAB	PITX1
	HGD	HIST1H4J
	NYX	REEP2
	SLITRK5	CALB2
	MYH14	APOC2
	SULT1C2	COL11A1
	ZSWIM5	
	SLC35F1	
	AVPR1B	
	PRLR	
	RAB19	
	OR9A4	
	ARHGAP42	
	HOOK1	
	PAIP2B	
	CDK18	
	UGT1A1	
	LHFPL3	
	PNPLA3	
	PDK2	
	TRIM74	
	FAM189A1	
	RCAN2	
	TMEFF2	

FMO2
SLC22A8
TMEM139
MPPED2
C10orf113
C1orf115
SMPDL3A
AC021218.2
AKAP6
ZNF395
BEX5
FRAS1
GAS2
FAAH
ZSCAN31
LIN7A
RGS5
TM4SF18
ACAA2
MACC1
WBSCR27
CIB4
CBLC
TLN2
MOGAT1
RNF152
CGN
CHST13
IHH
AMOT
C9orf66
FRMPD2
IQSEC3
NXNL2
SPATA31E1
EXPH5
TFPI
NOSTRIN
FN3K
CRYZ
HOXC10
APOLD1
TIMD4
C4A
UNC5CL
SOX6
CNNM1
ALDH4A1
PAQR5
NRG3
RAB3IP
DNAH6
SLC22A18
GJA4
UCN3
INSR
VCAM1
DGKB
LRRK2
DDAH1

RORC
AP1M2
SPINK7
KCNAB1
KLHDC7A
ACBD4
SPEF2
DPP4
HOXA4
PEBP1
ZNF711
KRBA1
SEMA6A
GLTPD2
SERINC2
BNIP3
CDH1
EDNRB
CDH6
HIST1H2AA
FLT1
ADH6
IL1RL2
C4orf19
LAMA1
EPB41L4A
LRRTM3
OXER1
TCN2
ZBTB16
BSPRY
HEY2
ARHGEF28
HYAL1
NEGR1
GLUD2
SNAP25
KLF8
C6orf223
TLL1
C2orf73
SLC14A1
TMEM200A
RIPK4
PEX11A
ERICH2
PIPOX
CXorf67
EFHC2
SLC22A4
TEX9
RAB11FIP3
GLB1L2
FRZB
HRC
C6
DZIP1
VEPH1
ABHD6
TTC38

	RHOBTB1
	LIPC
	SPATA18
	NDRG2
	AMACR
	UPP2
	AMIGO1
	C4B
	ALPK2
	CLDN3
	TRIM9
	SLC25A21
	PLA2G16
	SERPINI1
	KIAA1549
	FAM149A
	AC092687.4
	RBP7
	SPINK9
	ENTPD2
	NUP62CL
	MARVELD3
	CGREF1
	TMEM38A
	ACOX2
	ZC2HC1C
	TEF
	PCSK6
	PLEKHA7
	CREB5
	TMEM159
	RASSF6
	UGT8
	CCDC148
	PPP1R17
	OR51B4
	AIG1
	F8
	NLRP6
	CSPG4
	NOVA1
	CNBD2
	SLC2A9
	SLC16A4
	SPATA6L
	ACSM3
	LIFR
	SLC44A3
	IL1RAPL2
	EFNA1
	NMNAT3
	MUC15
	SPPL2C
	AC129492.6
	CCDC110
	LRIG3
	TMEM176B
	ATP7B
	ENPP2
	ACTR3C

ANK3
TEKT3
CDKL2
SCN4B
CYP4V2
NFIA
HSPB8
RP4-539M6.19
MTFP1
SBSPON
PARD6B
LRRC66
ILDR2
ABI3BP
CTC-236F12.4
SCN1A
FAM65C
FAM134B
PRELID2
EFCAB12
TBPL2
CA2
PLSCR2
HSD17B3
FGF14
ENOSF1
FAHD1
ATP11A
RBM24
ZNF208
CXXC4
CX3CL1
FHL5
GDF7
OLFML2A
PRKAA2
SEMA4G
GRAMD1C
NEBL
PTGR2
ANXA4
TTC22
NGEF
ENPP5
MEP1B
BEND5
KCNF1
NOTCH4
RBKS
RNF208
DCHS2
NDRG1
SORBS1
ALDH6A1
ADM
TBX2
SLC25A42
SLC23A1
ZYG11A
OGDHL

	POU3F3
	FAM110C
	CYP4A22
	ATOH8
	DECR2
	NXF5
	PMM1
	RASD1
	NCR3LG1
	RGL3
	FHL1
	CIDEB
	ZNF804B
	HIPK2
	CHRM3
	GABRD
	SLC16A11
	EFCAB6
	FABP3
	PLA1A
	C5orf49
	ALDH3A2
	FGFR1OP
	TRABD2B
	AUTS2
	RNF128
	SYDE2
	POU5F1B
	AK7
	TNS1
	GDF15
	PRSS37
	MUC4
	CCND1
	SEC16B
	GOT1
	C4orf47
	ALDH5A1
	ANKRD33B
	RP11-307N16.6
	PLVAP
	PXMP2
	NTN4
	TMEM133
	RAG1
	MLYCD
	HUNK
	SLC39A14
	BCL2
	LRRC19
	FZD4
	KLHL23
	PRR26
	PDE7B
	LINGO4
	CBX7
	COL4A4
	EFHD1
	AGBL3
	DLL4

	KANSL1L
	CDH20
	TNFAIP6
	MYOM3
	TMEM232
	TSPAN18
	HOGA1
	PTPRM
	TMEM246
	TBC1D8B
	NTRK3
	CDH2
	ACSS3
	FOXQ1
	MOB3B
	NR3C2
	MOCS1
	ANKS1B
	CLDN7
	HNMT
	DSEL
	RBPMS2
	ARHGEF33
	ARHGEF16
	OCA2
	LOXHD1
	XYLB
	GFRA1
	SSTR1
	OR51Q1
	NRCAM
	SENP8
	CES2
	CRADD
	WDR31
	FGFR4
	DOK6
	CAPZA3
	ECH1
	CLYBL
	RNF148
	XKR9
	FRK
	A4GNT
	AC009403.2
	CLU
	AKR1C3
	CLEC4F
	SCARB1
	HSD17B14
	SLC9A3R1
	SLC6A8
	IFIT1B
	NIPSNAP3B
	PARK2
	SLC28A2
	NRG1
	EPB41L1
	TDRP
	NR1D1

	SOX13
	USP2
	COL21A1
	ABAT
	CLIC4
	PKP2
	KCNK5
	BDNF
	UBA5
	PPP1R32
	PTER
	ASPRV1
	DCDC1
	ECHDC2
	PPP2R3A
	GRM8
	ATP1B1
	SOWAHA
	ACADM
	SGK2
	SLC16A13
	FAM50B
	ABHD1
	PTGR1
	MTCP1
	GSTM4
	SPAG17
	CYFIP2
	HPCAL1
	LDHD
	ASTN2
	WWC1
	FGFR3
	DIRC3
	PPP1R3C
	LRRC36
	NIPSNAP1
	LGR4
	CDCA2
	SH3YL1
	PDZK1IP1
	TBX6
	ADHFE1
	RIBC1
	MAP3K7CL
	DHODH
	STAP2
	SPATA6
	SEC14L6
	SCN9A
	ITGA6
	NTRK2
	HES5
	EXOC3L2
	BIVM
	PLCB1
	ARHGEF37
	MICAL3
	RAB36
	FAM213A

	MAP3K13
	HEYL
	LEAP2
	ABCC3
	PLEKHG6
	RHOB
	EPS8
	PLSCR4
	IFT88
	ZNF69
	TM7SF3
	ACOT4
	SH3BGRL2
	DGCR6
	HRH2
	DBP
	VWF
	ACAT1
	TDRD6
	UBQLN3
	CREB3L4
	EBF2
	COBL
	CDK20
	TNFRSF11B
	LRMP
	SLC2A11
	PABPC4L
	STC2
	HSD17B8
	DDO
	FAM160A1
	EPS8L2
	NSUN7
	DENND6B
	LGALSL
	RDH13
	MACROD1
	SRL
	MTHFS
	ZNRF3
	PNMA2
	PGRMC1
	DNM3
	ANO2
	BCL7A
	THSD7A
	POPDC2
	KBTBD3
	NPHP1
	THRB
	MGST1
	FAM83G
	DPY19L2
	CNKSR3
	PDE9A
	UBXN10
	KDR
	KCNN3
	APITD1

AK4
MIPOL1
CTNNA3
CD36
L2HGDH
PRRG4
CHCHD10
TST
NOXA1
FGGY
KIAA1671
PRKAB1
BLOC1S5
RORA
SLC25A4
DAB2
TBL1X
SCD
ACY1
LEPROTL1
ACKR4
HAUS7
SPTLC3
ALDH3A1
PPIL6
CDS1
PROS1
ENTPD5
HSPA4L
IL17RD
ITGA8
GSTM2
DNAJB9
TNFRSF19
FSIP2
DNAH7
SLC25A27
ADAMTSL3
ISOC2
ISPD
GLRX
PPARA
SLC25A23
MAP2
GRTP1
CYB5R3
KIAA1456
RIT1
A4GALT
CPNE8
ZBTB20
DYNC2LI1
SPRY4
CAMSAP3
HEY1
RHBDL1
ASB13
NRIP2
CALML4
SLC5A4

SPR
ASS1
SDPR
PECR
ABCG2
HIBADH
MAPK8IP1
KHDRBS3
CXorf36
PIH1D2
SPINT2
SPEF1
SPARCL1
CLCN4
ANGPT2
FTO
RNF5
HOXD9
MPI
CACNA1D
CLK4
ACACB
ITGA1
PER2
CFI
TMEM135
FNBP1L
KLC4
IQCA1
MROH7
PDIA5
BDH2
TINAGL1
FLRT1
CSRNP3
KLHDC2
GDPD1
CTAGE5
MAPK10
FMO4
SHROOM1
CRY2
GSTA4
OCIAD2
ZNF608
CNNM2
PHOSPHO2
FAM13A
SEPSECS
DHRS4
DUSP1
KSR1
SEZ6L2
RAVER2
CDON
IL18R1
SPAG16
BCKDHA
SPIRE1
PDE8A

	TSNAXIP1
	CCDC30
	FGFR2
	TMEM143
	ZMAT1
	PTPRK
	FAH
	MSRA
	METTL9
	EPB41L5
	FBXO3
	LRRC23
	SHANK3
	EGFR
	HMGN3
	GPR4
	TNFRSF21
	GALNT15
	AKR7A2
	NUDT7
	APBB1
	GAS2L3
	TMEM14A
	PGPEP1
	TMEM192
	TP53INP2
	PARM1
	TMEM63A
	ANKAR
	ACSS1
	WDR91
	BPHL
	PAQR7
	TSGA10
	DNALI1
	HDAC11
	PLEKHA1
	ARHGAP24
	ZCCHC16
	RGL1
	RGS14
	PPM1L
	PITPNC1
	CPEB4
	TNIP1
	DHTKD1
	PEX7
	KIAA0895L
	ZNF366
	MTMR11
	APLF
	TTC21B
	LRGUK
	RBM47
	HIBCH
	CAMK2N1
	ACO2
	YPEL2
	FLT4
	GKAP1

	ENO1<br>PCCA<br>ZNF396<br>DOK4<br>DLL1<br>SLC22A17<br>NOVA2<br>GPRC5C<br>AIFM1<br>IQCK<br>HS1BP3<br>NBEA<br>SUSD2<br>SETD3<br>TAPT1<br>FBXL5<br>ARHGAP32<br>EZR<br>PIGH<br>MOAP1<br>MIF<br>ZNF697<br>CLMN<br>PLCL1<br>NIT2<br>CYP7B1<br>GUCY1A3<br>CC2D2A<br>SCP2<br>ADAM22<br>NUDT12<br>MYO7A<br>NDFIP2<br>MORN2<br>GPER1<br>STXBP1<br>AGPAT5<br>LPIN3<br>NOL3<br>TMEM140<br>TGFA<br>DNPH1<br>TMEM184A<br>MYCT1<br>STON2<br>FILIP1<br>CABLES1<br>SGCB<br>TTC28<br>ACADSB<br>PHYH<br>RDH5<br>ANKRD46<br>NHEJ1<br>WDR54<br>ACADS<br>TPCN2<br>AK9<br>PPFIBP1<br>ANKRD13A

	PER3
	QTRT1
	FZD1
	L3MBTL4
	ITM2B
	DYNC2H1
	PLCH1
	MAF
	CEP70
	PCBD2
	CGNL1
	PKIG
	ARVCF
	EXOC3
	CCDC89
	SLC26A1
	RTKN
	GHDC
	BEND7
	RASGRP3
	LMTK2
	RAPGEF4
	CDKL1
	SSPN
	UNC119B
	CRIM1
	ETFDH
	NEDD9
	ZDHHC1
	NETO2
	INO80C
	DNAH14
	ARHGAP6
	ARHGAP33
	D2HGDH
	CLEC14A
	FBXO21
	PNPLA4
	AJUBA
	GCSH
	PPP1R13L
	GALNT11
	SPECC1L-ADORA2A
	RBPMS
	LPCAT3
	LRP5
	MMAB
	THRA
	PRPSAP2
	NDUFA5
	RCBTB2
	MYO15B
	LMBRD1
	MXI1
	KANK1
	DARS
	TTLL7
	HES1
	CYP4X1
	CERS4

BBS12
RAI2
FZD5
CACHD1
AFAP1L1
HADHB
CCDC28A
ST13
TMEM205
CYSTM1
SEMA6C
ARHGEF17
BCAM
TSPAN9
C17orf97
TRIM66
ABCA5
PCYOX1
ALG12
CARD10
C17orf107
IFT46
ALDH7A1
SUOX
SOD2
EPHA4
PEAR1
ZNF483
STOX2
HCN3
PCLO
NEDD4L
MRPL40
IFT172
CCNB1IP1
DENND1C
CD200
NREP
SPRY1
NFIB
RCAN1
KLF10
SDC4
NRARP
B3GALNT1
TNK1
TMTC2
KAZN
ARSB
SIK2
KRT18
ZNF737
EHBP1
RP11-166B2.1
CPEB2
CYB5D2
MOK
SLC15A4
ARMCX4
PKP4

BNIP3L
FUNDC2
PTPRB
NET1
TXNDC16
HIP1R
HOOK2
TSPAN6
ISOC1
ABCB6
TMTC3
CBY1
ZC3H12B
NEK11
MAPRE2
SAP18
TRIB2
TMEM150A
KIAA1958
MAML2
LNX1
RAPGEF2
WDR78
UNC13B
RAB21
ARHGAP26
PRKD1
ZNF433
TNFRSF14
RAB11FIP5
IVNS1ABP
ADCY9
CAT
FUZ
DMTN
CCDC7
TOM1L1
DLG3
AUH
PINK1
ANKRD42
PIK3CB
NT5DC1
TTC12
PRKG1
DTWD2
ACOT2
CRIPAK
MCAT
JAG1
SIRT3
ZNF470
PGK1
CPT2
SNX29
SLC12A7
PAPLN
TULP3
MGST2
DHRS12

	ZNF189
	SPATA7
	FARP1
	KIAA1147
	METAP1D
	TUBE1
	OSBPL1A
	GJC1
	GRAMD3
	SARM1
	PLEKHF2
	NQO2
	ACLY
	GUCY1B3
	CNNM3
	GTF2IRD2B
	GRB10
	GIT2
	ID2
	ZNF358
	CLUAP1
	STX3
	MPC1
	BIVM-ERCC5
	SDHA
	CCHCR1
	UBE3D
	ACADVL
	WDR13
	PDK1
	AAMP
	MYO9A
	COQ7
	PHYKPL
	SYNM
	GCNT2
	ARRDC2
	RAPGEF5
	SHROOM4
	TMCC3
	MTFR1
	CLN5
	MVB12A
	TMEM67
	LNX2
	RHPN2
	C20orf96
	RSPH3
	TPI1
	PTPRG
	TOLLIP
	HDAC6
	TMEM106A
	ECSIT
	N4BP2L1
	SFXN1
	CNPPD1
	GAB1
	SHMT2
	MVK

LCA5
PM20D2
SCRN3
CPT1A
WLS
CDK14
PHC1
SEPTIN10
ALDH9A1
VPS13A
TXNRD2
PLEKHA6
MLEC
CTDSPL
HAGH
TPMT
CLSTN3
ZNF462
ZNF611
HSDL2
DGCR6L
ZNF429
ZNF420
EPOR
ZDHHC9
ZFAT
ZHX3
PTPN3
DIXDC1
LRRC28
FGD4
KLF9
SLC43A2
PFKL
ALDOA
ZNF440
EIF4EBP2
NUMB
ABCD3
TCTN1
ETFA
LAPTM4A
GAS8
HSPA9
KIAA1107
BBS1
SLC2A13
DDT
TCP11L1
MCTP2
NFU1
ESD
ZNF160
PES1
UBXN2B
DNHD1
HDHD3
OARD1
ARHGEF12
GMCL1

	DCTN6
	MAPK1
	PMVK
	DOCK6
	AFG3L2
	NT5C3B
	GBA2
	WRB
	NADK2
	ALDH2
	NHLRC3
	CDK10
	SIAE
	CFDP1
	LYPLAL1
	NDUFC1
	TGDS
	ZBTB10
	AKAP1
	MTRR
	ARHGEF7
	ETFB
	IFT140
	GALNT18
	HADHA
	RETSAT
	CPSF7
	MRPS35
	SLC25A1
	TJP2
	CERS2
	WDR60
	DISP1
	SLC25A45
	ANKZF1
	ECI2
	ADI1
	PHLDB2
	COMMD1
	TCF7L2
	INPP5K
	WDR35
	ZSCAN2
	CLPTM1L
	LRP6
	ERVW-1
	ZNF397
	SLC25A11
	WWOX
	ITFG2
	WBP1
	CDK19
	SYNE2
	PDE4A
	PWWP2A
	CIRBP
	SUCLG1
	FITM2
	ST7
	TUBG2

	GPI
	VRK3
	CLSTN1
	MICU2
	DNAJB2
	MYO6
	ARHGEF10L
	MARCHF6
	DMXL1
	PAAF1
	DENND5B
	SLC35B4

Table S4. Clinical features of each subtype.

Variables	Subtype 1 (n = 60)	Subtype 2 (n = 75)	Subtype 3 (n = 38)	*p*-value
Median (range), years	**57 (18–85)**	**61 (42–88)**	**60 (30–82)**	*.028*
Sex (N, %)				
Male	**14 (21.9)**	**21 (28.0)**	**10 (26.3)**	*.703*
Female	**50 (78.1)**	**54 (72.0)**	**28 (73.7)**	
IMDC risk (N, %)				
Favorable	**17 (28.8)**	**23 (33.8)**	**9 (26.5)**	
Intermediate	**30 (50.8)**	**38 (55.9)**	**15 (44.1)**	*.201*
Poor	**12 (20.3)**	**7 (10.3)**	**10 (29.4)**	
ORR (N, %)				
CR	**2 (3.1)**	**1 (1.3)**	**1 (2.6)**	
CR/PR	**4 (6.2)**	**6 (8.0)**	**5 (13.2)**	
PD	**30 (46.9)**	**28 (37.3)**	**16 (42.1)**	*.573*
PR	**11 (17.2)**	**11 (14.7)**	**4 (10.5)**	
SD	**16 (25.0)**	**23 (30.7)**	**9 (23.7)**	
Unknown	**1 (1.6)**	**6 (8.0)**	**3 (7.9)**	
Benefit (N, %)				
CB	**22 (34.4)**	**23 (30.7)**	**13 (34.2)**	
ICB	**12 (18.8)**	**21 (28.0)**	**9 (23.7)**	*.584*
NCB	**30 (46.9)**	**29 (38.7)**	**16 (42.1)**	
Unknown	**0 (0.0)**	**2 (2.7)**	**0 (0.0)**	

Abbreviations: IMDC, International Metastatic RCC Database Consortium; ORR, objective response rate; CR, complete response; PR, partial response; SD, stable disease; PD, progressive disease; CB, clinical benefit; ICB, intermediate clinical benefit; NCB, nonclinical benefit.
